# Comparative evaluation of multi-fold rib and structural iliac bone grafts in single-segment thoracic and thoracolumbar spinal tuberculosis: clinical and radiological outcomes

**DOI:** 10.1186/s13018-023-04416-x

**Published:** 2023-12-01

**Authors:** Yuanrui Luo, Hua Chen, Peng Xiu, Jiancheng Zeng, Yueming Song, Tao li

**Affiliations:** Department of Orthopedics, Orthopedic Research Institute, West China Hospital, Sichuan University, No. 37 Guo Xue Rd, Chengdu, China

**Keywords:** Autogenous bone grafts, Thoracic and thoracolumbar, Spinal tuberculosis, Surgery

## Abstract

**Objective:**

To compare clinical and radiological outcomes of multi-fold rib and structural iliac bone grafts, the primary autologous graft techniques in anterolateral-only surgery for single-segment thoracic and thoracolumbar spinal tuberculosis.

**Methods:**

This retrospective study included 99 patients treated from January 2014 to March 2022, categorized into 64 with multi-fold rib grafts (group A) and 35 with structural iliac bone grafts (group B). Outcomes assessed included hospital stay, operation time, intraoperative blood loss, postoperative drainage, complications, erythrocyte sedimentation rate (ESR), C-reactive protein (CRP), the Visual Analog Scale (VAS) for pain, the Oswestry Disability Index (ODI), bone fusion time, and the American Spinal Injury Association (ASIA) impairment scale grade. Segmental kyphotic angle and intervertebral height were measured radiologically before surgery and follow-up.

**Results:**

The mean follow-up was 63.50 ± 26.05 months for group A and 64.97 ± 26.43 months for group B (*P* > 0.05). All patients had achieved a clinical cure. Group A had a shorter operation time (*P* = 0.004). Within one week post-surgery, group B reported higher VAS scores (*P* < 0.0001). Neurological performance and quality of life significantly improved in both groups. No significant differences were observed in segmental kyphotic angle and intervertebral height between the groups pre- and postoperatively (*P* > 0.05). However, group A showed a greater segmental kyphotic angle at the final follow-up, while group B had better maintenance of kyphotic angle correction and intervertebral height (*P* < 0.05). Bone fusion was achieved in all patients without differences in fusion time (*P* > 0.05).

**Conclusions:**

Multi-fold rib grafts resulted in shorter operation times and less postoperative pain, while structural iliac bone grafts provided better long-term maintenance of spinal alignment and stability, suggesting their use in cases where long-term outcomes are critical.

## Introduction

Spinal tuberculosis (STB) represents the predominant form of skeletal tuberculosis, accounting for nearly 44% of skeletal cases and making up about 10% of all extrapulmonary tuberculosis (TB) instances [1, 2]. The consequences of STB are severe, with the disease frequently affecting the intervertebral disks and adjacent vertebral body end plates, leading to complications such as kyphotic deformity and potential paraplegia. Although standard chemotherapy yields positive outcomes in a significant majority (82–95%) of STB patients, surgical intervention is required for those presenting with neurological deficits, large cold abscesses, recurrent infections, severe pain, local kyphosis, or segmental instability [3–7]. Classic surgical treatment for STB involves anterolateral tuberculosis foci debridement and interbody bone grafting, providing direct lesion access for thorough removal and alleviation of spinal cord compression, a benefit not as readily achieved with posterior surgeries [8]. However, the stability provided by spinal screw systems is temporary, with long-term stability dependent on successful vertebral bone fusion. While allogeneic bone grafts and titanium mesh cages are common, they carry risks such as reduced osteoinductive potential, lack of osteoprogenitor cells, immune reactions, and a slight risk of disease transmission [9–12]. In contrast, autologous rib and iliac bone grafts used in anterolateral-only STB surgeries are preferred for their osteogenic, osteoconductive and osteoinductive properties and their biocompatibility [13]. This study aims to fill the gap in the literature by providing a detailed comparison of the clinical and radiological outcomes associated with these autologous bone grafts in the treatment of thoracic and thoracolumbar STB.

## Materials and methods

### Patient selection

This research received approval from the Ethics Review Committee of West China Hospital, Sichuan University. All participating patients provided written informed consent.

Inclusion criteria: (1)pathological confirmation of TB diagnosis located in the thoracic and thoracolumbar (T1-L1); (2)tuberculosis foci confined to a single vertebral segment, defined as the involvement of a single vertebral body or an intervertebral disc along with its immediately adjacent superior and inferior vertebrae; (3) patients underwent anterolateral-only surgical debridement, internal fixation, and reconstruction utilizing autologous bone grafts (either iliac or rib).

Exclusion criteria: patients with a confirmed diagnosis of active pulmonary or extrapulmonary TB, malignancy, discontinuous or multi-segmental STB, osteoporosis, recent traumatic fractures, or those who had thoracic and thoracolumbar surgeries within the past six months were excluded.

Study Cohort: data from 99 patients diagnosed with thoracic and thoracolumbar TB between January 2014 and March 2022 were retrospectively analyzed. These patients underwent surgical debridement of tuberculosis foci, instrumentation, and interbody fusion with autologous bone grafts via an anterolateral-only surgical approach. The cohort was divided into group A (64 patients who received autogenous multi-fold rib bone grafts) and group B (35 patients given autogenous structural iliac bone grafts).

Patients predominantly presented with clinical symptoms such as back pain, motor weakness, mild fever, and varying degrees of lower limb dysfunction. Elevated ESR and CRP levels were observed across patients. Preliminary diagnosis was established using spinal X-rays, computed tomography (CT), and magnetic resonance imaging (MRI) results that depicted vertebral bone erosion, reduced or vanished intervertebral spaces, and cold abscesses.

### Preoperative management

Upon suspecting a clinical diagnosis, all patients were promptly initiated on standard anti-tuberculosis chemotherapy. The HREZ regimen was adopted, comprising isoniazid (5 mg/kg/day, max 300 mg/day), rifampicin (10 mg/kg/day, max 1200 mg/day), pyrazinamide (30 mg/kg/day, max 2000 mg/day), and ethambutol (15 mg/kg/day, max 2500 mg/day). This treatment commenced 3–4 weeks before surgery to reduce bacterial burden and inflammation, optimizing surgical conditions. Surgery was primarily deferred until ESR fell below 40 mm/h, and CRP levels consistently declined. However, in cases of progressive paralysis, surgery was expedited to prevent further neurological damage. Concurrently, supportive nutritional interventions were implemented to address underlying hypoproteinaemia and anemia.

### Surgical procedure

After administering general anesthesia, patients were laterally positioned with the more affected side upwards. An anterolateral incision exposed the affected vertebrae and corresponding rib, which was then sectioned. Comprehensive debridement of the diseased area was executed. Post-debridement, spinal defects were assessed and corrected using multi-fold rib bone grafts in Group A (as per Hodgson and Stock [14, 15])or structural iliac bone grafts in Group B (based on established literature [16]). A lateral screw fixation system was routinely employed for spinal further stabilization. In cases where vertebral destruction was significant, the fixation was extended to the adjacent normal vertebrae to enhance stability. The surgical site was irrigated with sterile saline infused with streptomycin (1 g), followed by drainage tube placement and dispatch of samples for pathological examination.

### Postoperative care

The drainage tube was extracted after the drainage volume reduction to below 50 ml/day. After surgery, patients were required to continue oral anti-tuberculosis medication for a total treatment duration of no less than 18 months. Initiatives for postoperative rehabilitation commenced three days after the surgical intervention.

### Clinical assessments

For all cases, the indexes were recorded preoperatively, postoperatively within one week, every three months during the first year after surgery, every six months in the second year, and at FFU (final follow-up). The assessment spanned various metrics, from surgical specifics like the duration of the operation, hospital stays, operation time, and volume of blood loss to routine lab tests monitored markers such as Complete Blood Count (CBC), ESR, CRP, and the functions of the liver and kidneys. Neurological function was evaluated using the ASIA scale, while the ODI was employed to assess the impact on patients’ quality of life. Pain intensity was quantified with the VAS, covering assessments of back pain, radiating pain in the lower extremities, and pain at the graft donor site.

### Radiological assessments

Following surgery, patients were subjected to detailed radiological evaluations at intervals matching their clinical follow-up schedule to evaluate for any recurrence of spinal tuberculosis, assess bone healing, and check the stability of the spine and internal fixation. These assessments included precise measurements of intervertebral height and segmental kyphotic angles. Intervertebral height was defined as the distance between the upper and lower endplates of the vertebral bodies within the coronal plane of the fused segment, as per the method described in reference [17]. Segmental kyphotic angle was determined by the angle between the upper endplate of the vertebra proximal to the lesion and the lower endplate of the vertebra distal to the lesion. The Bridwell et al. criteria [18] were employed to determine bone graft fusion, with X-ray and CT scans utilized as appropriate. All imaging data underwent review by an experienced spine surgeon and a senior radiologist.

### Statistical analysis

Statistical evaluations were performed utilizing the SPSS software. (version 22.0, Chicago, IL, USA). Differences between the study groups were analyzed using the *t*-test for independent samples and Chi–Square and Mann–Whitney *U* tests when applicable. For the analysis of pre-and post-treatment effects within the same group, the paired *t*-test was utilized. Statistical significance was established at a *P*-value threshold below 0.05.

## Results

### Clinical assessments

**Fig. 1:**
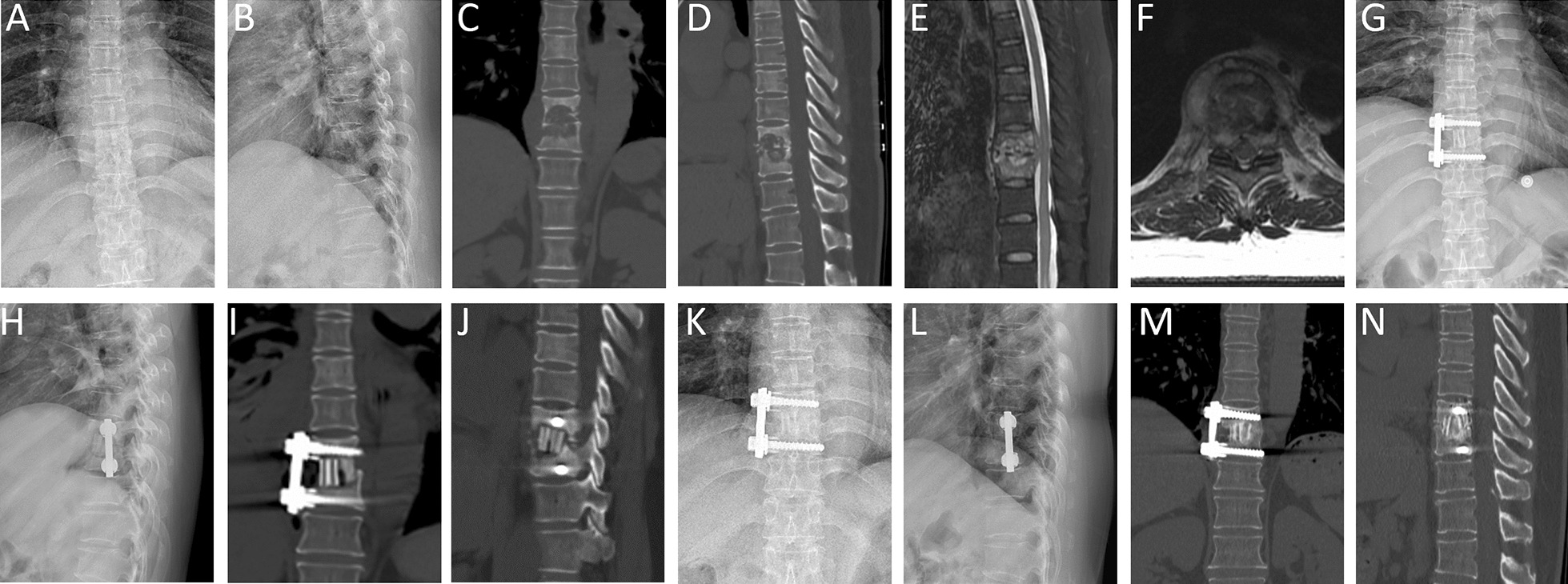
A 53-year-old woman with T9–10 tuberculosis. **A**, **B** The X-ray film before surgery demonstrated intervertebral stenosis between the T9–10. **C–F** Preoperative CT and MRI showed T9–10 vertebral bone destruction with paravertebral and intraspinal abscess. **G–J** Postoperative X-ray and CT of the patient who underwent anterolateral debridement and instrumentation at T9–10 with autologous rib bone graft. **K–N** X-ray and CT at 24 months postoperative showed good bone fusion between T9–10, without signs of tuberculosis recurrence and no apparent bone absorption or fractures

**Fig. 2:**
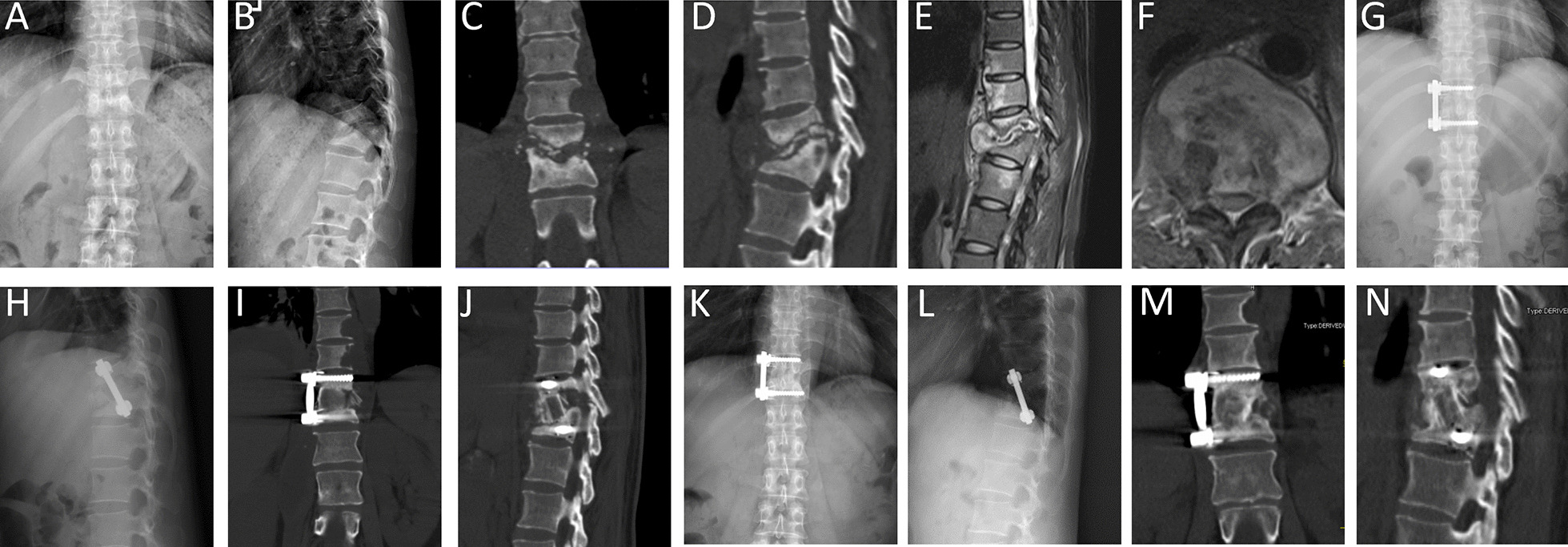
A 45-year-old woman with T11–12 tuberculosis. **A**, **B** Preoperative X-ray before surgery demonstrated vertebral bone destruction and kyphosis at T11–12. **C–F** Preoperative CT and MRI showed T11–12 vertebral bone destruction with paravertebral and intraspinal abscess. **G–J** Postoperative X-ray and CT of the patient underwent anterolateral debridement and instrumentation at T11–12 with autologous structural iliac bone graft. **K–N** X-ray and CT at 25 months postoperative showed good bone fusion and spinal alignment without signs of tuberculosis recurrence and hardware failure

A total of 99 patients were included in the study, with 64 in group A receiving multi-fold rib grafts (Fig. 1) and 35 in group B receiving structural iliac bone grafts (Fig. 2). The groups were followed for a mean duration of 63.50 ± 26.05 months (group A) and 64.97 ± 26.43 months (group B), with no statistically significant difference in follow-up times (*p* = 0.79). Demographic and baseline clinical characteristics, including sex distribution (*p* = 0.481) and age (*p* = 0.158), were comparable between the groups (Table 1). Similarly, no significant differences were found when comparing initial and final follow-up ODI scores (*p* = 0.730 and *p* = 0.486, respectively) and VAS scores for pain (*p* = 0.600 and *p* = 0.655, respectively) (Table 2). The amount of intraoperative blood loss (*p* = 0.687) and length of hospital stay post-surgery (*p* = 0.560) were also similar between the two cohorts (Table 1). ESR and CRP levels did not differ significantly at any measured time point pre- and post-surgery between the groups (Table 2).

**Table 1:** Basic clinical data and evaluation indexes comparison of each group

Item	Group A(64)	Group B(35)	*P* value
Gender (Male/Female)	30/34	19/16	0.481
Age(years)	43.32 ± 14.82	47.90 ± 16.21	0.158
Hospital stays (days)	13.89 ± 6.04	13.23 ± 3.86	0.560
Duration of follow-up (months)	63.50 ± 26.05	64.97 ± 26.43	0.790
Operation time (min)	229.56 ± 45.64	255.51 ± 32.95	0.004
Operation blood loss (ml)	335.16 ± 157.03	349.14 ± 177.31	0.687
Postoperative drainage (ml)	610.31 ± 604.15	704.29 ± 338.89	0.398

**Table 2:** Comparison of clinical parameters between the two groups

Item	Group A(64)	Group B(35)	*P* value
ESR preop (mm/h)	48.56 ± 27.250	43.02 ± 19.48	0.290
ESR 3 months postop (mm/h)	13.81 ± 8.61	17.13 ± 8.37	0.067
ESR final follow-up(mm/h)	6.11 ± 2.63	6.00 ± 2.80	0.847
CRP preop(mg/L)	28.81 ± 30.55	21.76 ± 23.23	0.237
CRP 3 months postop(mg/L)	4.23 ± 2.21	4.55 ± 2.23	0.505
CRP final follow-up(mg/L)	2.59 ± 1.23	2.97 ± 1.10	0.133
ODI preop	52.14 ± 15.06	53.20 ± 13.56	0.730
ODI final follow-up	9.41 ± 3.85	8.89 ± 2.88	0.486
VAS preop	6.16 ± 0.37	6.11 ± 0.40	0.600
VAS 1 week postop	3.80 ± 0.443	4.63 ± 0.84	< 0.0001
VAS final follow-up	2.02 ± 0.13	2.03 ± 0.17	0.665

*ESR* Erythrocyte sedimentation rate, *CRP* C-reactive protein, *ODI* Oswestry disability index, *VAS* Visual Analog Scale, *Preop* Preoperation, *Postop* Postoperation

However, group B experienced a longer operation time and higher average VAS scores within the first postoperative week, indicating more significant pain compared to group A (*p* < 0.05, Tables 1 and 2). Neurological function improved in all but one patient in each group, who maintained their preoperative neurological status at the final follow-up (Table 3).

**Table 3:** ASIA grade for neurological status valuation

Groups	Cases	Grades	Preoperative	Last follow-up			
				B	C	D	E
A	64	A	2	1	1		
		B	4		4		
		C	5		1	4	
		D	26				26
		E	27				27
B	35	B	2		1	1	
		C	8			8	
		D	11			1	10
		E	14				14

### Radiological assessments

Radiological assessments confirmed that all patients achieved clinical cure at the final follow-up. The average time to bony fusion was 7.97 ± 2.34 months for group A and 8.17 ± 2.55 months for group B, with no significant difference between the groups (*p* = 0.271). There were no significant differences in the segmental kyphotic angle and intervertebral height before and after surgery within each group (*p* > 0.05). Kyphosis was corrected by an average of 7.55 ± 3.97 degrees in group A and 7.29 ± 3.95 degrees in group B, with intervertebral height restoration averaging 0.51 ± 0.45 cm and 0.54 ± 0.52 cm, respectively. Notably, at the final follow-up, patients in group A exhibited a larger segmental kyphotic angle and a more substantial loss in kyphotic correction and intervertebral height than those in group B, with these differences reaching statistical significance (*P* < 0.05, Table 4).

**Table 4:** Comparison of radiologic parameters between the two groups

Item	Group A(64)	Group B(35)	*P* value
Segmental kyphotic angle preop (°)	20.68 ± 7.23	18.90 ± 7.84	0.271
Segmental kyphotic angle postop (°)	13.39 ± 6.56	11.35 ± 7.34	0.173
Segmental kyphotic angle final follow-up (°)	18.55 ± 7.46	14.74 ± 7.71	0.019
Segmental kyphotic angle correction (°)	7.29 ± 3.95	7.55 ± 3.97	0.751
Loss of correction angle (°)	5.15 ± 3.21	3.39 ± 3.13	0.009
Intervertebral height preop (cm)	4.13 ± 0.78	4.09 ± 0.31	0.803
Intervertebral height postop (cm)	4.64 ± 0.65	4.63 ± 0.58	0.956
Intervertebral height final follow-up (cm)	4.20 ± 0.71	4.41 ± 0.40	0.111
Intervertebral height correction (cm)	0.51 ± 0.45	0.54 ± 0.52	0.795
Loss of intervertebral height (cm)	0.44 ± 0.30	0.22 ± 0.35	0.002
Bone fusion time (m)	7.97 ± 2.34	8.17 ± 2.55	0.271

### Complications

Complications were minimal and effectively managed in both groups. In group B, one patient developed a superficial wound infection, which responded well to prompt debridement, suturing, and targeted antibiotic therapy. In group A, there was an instance of incisional fat liquefaction, successfully resolved through daily enhanced wound dressing changes. Both groups had one case of STB recurrence, the one in group B developing a sinus tract at the surgical site; these two were effectively treated with debridement and catheter drainage. Additionally, four patients in the study developed postoperative pleural effusion, which spontaneously resolved within three months. Six patients experienced drug-related complications, which were successfully managed with hepatoprotective agents or urate-lowering medications (Table 5).

**Table 5:** Comparison of the postoperative complications in Groups A and B

Complications	Group A	Group B
Sinus formation	0	1
Superficial wound infection	1	0
Incisional fat liquefaction	1	0
Tuberculosis recurrence (re-formation of cold abcess)	1	1
Hydrothorax	3	1
Drug-related complication	4	2
Pain in bone extraction area	6	7

## Discussion

Spinal tuberculosis (STB) is the predominant form of extrapulmonary tuberculosis, comprising half of the cases of musculoskeletal TB. It primarily affects the anterior and middle columns of the spine, which can lead to severe outcomes, including vertebral collapse, cold abscesses, kyphotic deformity, and neurological deficits, with extensive socio-economic repercussions [18, 19]. The cornerstone of STB management is chemotherapy, utilizing drugs such as isoniazid, streptomycin, ethambuto, and ofloxacin, which are selected for their ability to penetrate abscesses and achieve effective concentrations [20–22]. Nevertheless, surgical intervention becomes indispensable for patients presenting with conditions such as kyphotic deformities or significant neurological deficits, aiming to eradicate the infectious foci, restore nerve functions, and approve and maintain spinal alignment.

In thoracic and thoracolumbar TB, the vertebral anatomy, including the pedicle and lamina, forms a sealed cavity particularly susceptible to disease progression due to its limited spinal canal space and poor vascularization. The accumulation of TB-induced necrosis, pus, and debris within this cavity can severely compress the spinal cord, leading to vertebral collapse and an increased risk of paraparesis or paraplegia [23, 24]. According to Denis’ three-column theory [25], the integrity of the anterior and middle columns is vital for spinal stability, which is often compromised in spinal TB. Consequently, effective treatment requires not only the meticulous removal of the TB foci but also the restoration of structural integrity, typically through bone grafting after debridement. While allografts are convenient, they carry risks such as higher failure rates and the possibility of disease transmission. While effective as materials for filling bone defects and supporting spinal stability, titanium mesh cages come with the drawbacks of higher cost and being prone to subsidence [26–28]. Autologous bone grafts such as rib, fibula and iliac, are widely used for their osteogenic, osteoconductive and osteoinductive properties and their biocompatibility [7, 9–12, 29, 30]. Rib and iliac bone grafts are commonly used due to their osteoinductive properties. Still, they are not without complications, such as pain, hematoma, and infections at the harvest site [16, 31]. Our study addresses the paucity of direct comparisons between these two types of autologous grafts, providing a detailed comparison of their clinical and radiological outcomes in the surgical treatment of thoracic and thoracolumbar TB.

Rib bone grafts are known for their biological potential and ability to achieve rapid bone fusion [32–34], our study revealed that all patients who received the autogenous multi-fold rib graft achieved bony fusion within an average duration of 7.97 ± 2.34 months. A notable advantage of the rib graft is the absence of a need for an extra incision: the rib bone strut required for grafting is excised during routine anatomical dissection of the surgical procedure. Consequently, this approach reduces surgical trauma, shortens operative time, and may lessen postoperative discomfort. Our data demonstrated that the postoperative VAS within one week in group B was significantly higher than in group A. Moreover, securely fixing the multi-fold ribs together during surgery prevents early graft displacement, thereby enhancing contact with the graft bed. Furthermore, the graft’s dimensions can be intraoperatively adjusted to optimize support and maintain the postoperative kyphotic correction angle. Despite these benefits, a challenge that occasionally arises is the need to sever the intercostal nerve, elevating the risk of postoperative chest and intercostal discomfort. In our cohort, this was noted in six patients but was effectively managed with nonsteroidal anti-inflammatory drugs, showing marked improvement within three months post-surgery.

The autogenous structural iliac bone graft is widely recognized as the optimal choice for managing spinal tuberculosis, primarily due to its robust mechanical strength derived from its tri-cortical structure(33, 34). Though fusion rates in literature for iliac grafts show variation, they consistently report a rate exceeding 90% [35, 36]. In our investigation, patients who opted for structural iliac bone grafts attained bony fusion in an average of 8.17 ± 2.55 months, with commendable outcomes in segmental kyphotic angle and intervertebral height correction. Nevertheless, donor site morbidity remains a concern, with up to 40% of recipients experiencing complications such as pain, nerve damage, hematoma infection, or fracture [16, 37, 38]. Even if not always manifested, these concerns can lead to extended operational time, longer hospitalization, increased blood loss, and residual scarring, which are significant in both recovery and cosmesis. In our study, although severe complications like hematomas or infections were absent, seven patients from group B reported significant pain at the donor site, which was manageable with a sustained regimen of nonsteroidal anti-inflammatory drugs.

It is worth noting that our findings underscore slightly better preservation of segmental kyphotic correction and intervertebral height in patients receiving group B treatments, stressing that the success of bone grafts in spinal reconstruction transcends biomechanical attributes to encompass biological behavior, such as osteoconduction, osteoinduction, and osteogenesis [39–41]. Rib bone grafts, which include cortical and cancellous components, are advantageous in reconstructive surgery for their accessibility and malleability for defect accommodation. Nonetheless, the rib’s inherent structural features, such as reduced mechanical strength and a thinner cortical layer, may render it more susceptible to resorption or failure under spinal physiological stresses. Moreover, the osteogenic capacity of rib bone grafts may be comparatively lower than that of iliac grafts due to a relatively lower proportion of cancellous bone and a corresponding scarcity of osteoinductive factors, including bone morphogenetic proteins (BMPs) and growth factors [42–44]. Conversely, the iliac crest, with its abundance of cancellous bone, serves as a robust source of osteoprogenitor cells and vascular marrow, facilitating superior osteoinduction and a vigorous osteogenic response after implantation [40, 45–48]. Its denser cortical layer imparts enhanced resistance to compression, which is crucial for maintaining the stability of the anterior and middle columns post-surgery. Therefore, while rib and iliac bone grafts are both feasible for anterior column reconstruction in spinal tuberculosis, our research favors the iliac crest for its superior long-term stability and biological integration.

While our study offers valuable insights, several limitations merit consideration. The retrospective design and small sample size may introduce inherent biases. A larger cohort and extended follow-up durations would provide a more robust assessment—moreover, potential inconsistencies arising from biases and disparities between observers needed to be systematically addressed. Lastly, the study did not quantify the volume of bone grafts harvested from rib and iliac bone, as quantification could provide additional insight into the relationship between bone graft volume and fusion rate, which should be suggested as a consideration point for future research. However, our findings contribute to a nuanced understanding and perspective. In the future, the field would benefit from prospective, randomized studies with longer follow-up times to further validate our observations.

## Conclusions

Both bone graft procedures yielded comparable postoperative clinical outcomes in treating thoracic and thoracolumbar TB. Notably, the multi-fold rib bone graft had an edge in minimizing surgical discomfort, however, with less deterioration in segmental kyphotic angle correction and intervertebral height, the structural iliac bone graft emerged as a superior method for ensuring long-term spinal stability.

## Data Availability

The datasets used and/or analyzed during the current study are available from the corresponding author upon reasonable request.

## Abbreviations

TB: Tuberculosis
STB: Spinal tuberculosis
ESR: Erythrocyte sedimentation rate
CRP: C-reactive protein
CT: Computed tomography
MRI: Magnetic resonance imaging
ASIA: American Spinal Injury Association
ODI: Oswestry Disability Index
VAS: Visual analog score
HREZ: Isoniazid, rifampicin, ethambutol, pyrazinamide
